# Decreased Quality of Life in Individuals with Type 2 Diabetes Mellitus Is Associated with Emotional Distress

**DOI:** 10.3390/ijerph16152652

**Published:** 2019-07-25

**Authors:** Elena Gómez-Pimienta, Thelma Beatriz González-Castro, Ana Fresan, Isela Esther Juárez-Rojop, Miriam Carolina Martínez-López, Hugo Adrián Barjau-Madrigal, Iris Rubí Ramírez-González, Esteban Martínez-Villaseñor, Esther Rodríguez-Sánchez, Mario Villar-Soto, María Lilia López-Narváez, Carlos Alfonso Tovilla-Zárate, Alma Delia Genis-Mendoza

**Affiliations:** 1 División Académica Multidisciplinaria de Comalcalco, Universidad Juárez Autónoma de Tabasco, Comalcalco, Tabasco P.C. 86650, Mexico; 2 División Académica de Ciencias de la Salud, Universidad Juárez Autónoma de Tabasco, Villahermosa, Tabasco P.C. 86100, Mexico; 3 Subdirección de Investigaciones clínicas, Instituto Nacional de Psiquiatría Ramón de la Fuente Muñiz, Ciudad de México P.C. 14370, Mexico; 4 Hospital Civil de Guadalajara, Guadalajara, Jalisco P.C. 44280, Mexico; 5 Hospital de Alta Especialidad “Gustavo A Rovirosa Pérez”, Secretaría de Salud. Villahermosa, Tabasco P.C. 86020, Mexico; 6 Hospital General de Yajalón “Dr. Manuel Velasco Suarez”, Secretaría de Salud. Yajalón, Chiapas P.C. 29930, Mexico; 7 Instituto Nacional de Medicina Genómica (INMEGEN), Ciudad de México P.C. 14610, Mexico

**Keywords:** distress, quality of life, diabetes, Mexican population

## Abstract

*Background*: individuals with type 2 diabetes show emotional distress as they learn how to cope with the disease. The emotional distress increases the possibility of complications in these patients. The aims of the present study were to evaluate the impact of the emotional distress in the quality of life of individuals with diabetes, and to investigate the demographic and clinical characteristics associated with the emotional distress of living with diabetes in a Mexican population. Methods: a total of 422 Mexican individuals with type 2 diabetes were recruited from the outpatient Diabetes Clinic of the Hospital Regional de Alta Especialidad Dr. Gustavo A. Rovirosa of Villahermosa, Tabasco. Demographic and clinical characteristics along with quality of life (SF-36) were assessed in these individuals. The emotional distress of living with diabetes was measured using the 5-item Problem Areas in Diabetes. Patients were divided according to the presence of high or low distress. *Results*: we identified that 31.8% (*n* = 134) of patients presented high diabetes-related emotional distress. We observed that hepatic diseases as comorbidities (*p* = 0.008) and diagnosis of major depression (*p* = 0.04) are factors associated with the emotional distress of living with diabetes. These patients showed a reduced quality of life in all dimensions (*p* < 0.001); the most affected dimensions were physical role (*d* = 0.37) and general health (*d* = 0.89) showing lower scores in comparison with patients with low emotional distress. *Conclusions*: our results suggest that Mexican individuals with type 2 diabetes mellitus show high emotional distress living with the disease and have a decreased quality of life. Therefore, it is necessary to decrease factors associated with the high emotional distress of living with diabetes in patients with type 2 diabetes.

## 1. Introduction

Diabetes mellitus is a global health problem and it is among the 10 leading causes of death worldwide [1]. It has been estimated that around 425 million people in the world suffer from diabetes mellitus; about 8.8% range between 20 and 79 years of age and 79% of them are in developing countries such as Mexico [1,2]. Mexico has the fifth highest prevalence of diabetes mellitus in the world, with the disease affecting 12 million people. It is estimated that in the next years we will move to the fourth position with 21.8 million people with diabetes mellitus [3].

Diabetes is caused by a deficiency in the production and use of insulin, which brings several consequences, particularly an abnormal elevation of blood glucose [4]. Furthermore, hyperglycemia affects several systems in the organism, causing heart disease, nephropathies, neuropathies and retinopathies among others. Additionally, hyperglycemia also triggers psychiatric problems such as anxiety, depression and stress [5,6]. Diabetes mellitus complications can bring severe disability to patients, even reducing their life expectancy [7].

Diabetes mellitus is a non-communicable disease; and apart from affecting life expectancy, it also affects physical, mental, and emotional life areas of the person. Psychosocial factors such as beliefs about the disease, medical expectations before receiving specialized treatment [8], economic status and perception of the disease [9], act as the most preponderant factors related to emotional distress of living with diabetes [10,11]. A recent meta-analysis suggests the need of implementing psychological interventions for decreasing diabetes-related emotional-distress [12] in the multidisciplinary treatment of diabetes.

The quality of life in individuals with diabetes mellitus is another area highly impacted [13], as dealing with this pathology is a burden for people who suffer it. Some studies have reported that the quality of life in patients with diabetes is directly related to several factors such as the educational achievement of the patient, previous conception and attitudes toward the disease and treatment, as well as the related comorbidities [14,15]. Nevertheless, there are no current reports related to the impact of emotional distress on the quality of life of individuals with diabetes. Therefore, the main objective of the present study was to compare demographic features, diabetes clinical characteristics and quality of life between patients with high and low diabetes-related emotional distress.

## 2. Materials and Methods

### 2.1. Ethics Statement

The present study was approved by the Ethics and Scientific Committees of the Hospital Regional de Alta Especialidad Dr. Gustavo A. Rovirosa in Villahermosa city, Tabasco State, Mexico. All patients over 18 years of age who attended the Diabetes Clinic of the Hospital were invited to participate. After receiving a verbal explanation of the nature of the study, those individuals who expressed interest in participating were given a full explanation of the study; those who accepted to participate signed a written consent form. All patients participated voluntarily and were not remunerated for their participation in the study.

### 2.2. Participants

A total of 422 individuals with current diagnosis of type 2 diabetes mellitus (DM2) were included in the study. All participants had been evaluated on at least one occasion in the Diabetes Clinic by a specialist asserting the diagnosis. Patients with uncontrolled diabetes at the time of the study were also included. When the participant was illiterate, the self-report measures were read by the person or family member who usually looked after/would regularly help the patient. Generally, patients attend their consultations accompanied by a relative and therefore, no patients were excluded due to their inability to read these measures.

### 2.3. Assessment Procedure

Demographic features (i.e., age, years of education, current occupation, marital status) and some clinical features (i.e., illness evolution, medical and psychiatric comorbidities, current alcohol consumption and nicotine use, current treatment with hypoglycemic drugs and insulin) were obtained through a face-to-face interview with the patients and their relatives, and the information was verified using clinical records to avoid memory bias. Body weight and height were used to determine current body mass index (BMI) and patients were classified as normal weight, overweight or obese following the parameters proposed by the World Health Organization. Glycosylated hemoglobin (HbA1c) concentration was determined by the colorimetric method. According to the recommended cut-point value, patients with HbA1c < 6.5% were considered as having adequate diabetes control, and those with values ≥ 6.5% indicated a poor/inadequate control [16].

Quality of life was assessed using the Short Form-36 (SF-36) validated in Mexican population which comprises eight dimensions: physical functioning, role limitations due to physical health, role limitations due to emotional problems, energy/fatigue, emotional well-being, social functioning, pain and general health. The scores of each dimension were obtained (0–100), higher scores define a more favorable health-related quality of life [17,18].

The 5-item Problem Areas in Diabetes (PAID-5) was used to measure the emotional distress of living with diabetes. Items are scored on a 4-point Likert scale (0–4 representing from “not a problem” to a “serious problem”), higher scores represent greater diabetes-related emotional distress. We used the optimal cut-off score of ≥8 to categorize patients with high and low distress [19].

### 2.4. Statistical Analysis

All statistical evaluations were performed using the Statistical Package for the Social Sciences (SPSS, IBM, Armonk, NY, USA), version 21. Descriptive statistics of all variables were calculated. Demographic and clinical variables were tested for differences between patients with high and low emotional distress using Fisher exact test or independent sample t tests where applicable. G*Power V.3.1.9.2 statistical software (SAS Institute Inc., Cary, NC, USA) was used to calculate effect size (Cohen *d*) for the significant results from the comparative analyses and were interpreted as small (*d* = 0.2), medium (*d* = 0.5), and large (*d* = 0.8) [20]. Significance level for tests was set at 0.05.

## 3. Results

### 3.1. Demographic and Clinical Features

Of the 422 individuals with DM2, 69.7% (*n* = 294) were women, with a mean age of 54.8 years (S.D. = 12.8, range 18–88 years). More than half of the participants were married or living with a partner (61.1%, *n* = 258), dedicated to non-remunerated activities (unemployed 13.5%, *n* = 57; housewives 50.5%, *n* = 213; full-time students 2.4%, *n* = 10), the average level of schooling was 6.9 years (S.D. = 4.4, range 0–26 years).

Participants had an average DM2 evolution of 15.1 years (S.D. = 8.7, range 1–53 years), more than three quarters were overweight/obese (BMI ≥ 25; 78.9%, *n* = 333) and presented high mean levels of HbA1C (8.2, S.D. = 2.0; HbA1C ≥ 6.5: 80.6%, n = 340), indicating a high proportion of uncontrolled diabetes in this sample. Neuropathy was the most reported medical comorbidity (45.3%, *n* = 191), while major depression (10.9%, *n* = 46) was the most reported psychiatric one. All patients were under treatment at the time of the study, the majority were under insulin shots and oral medications (63.3%, *n* = 267) followed by a smaller number of patients who were taking only oral medications (29.9%, *n* = 126) or insulin shots (6.9%, *n* = 29).

Following the cut-off score of ≥8 in PAID-5, we observed that 31.8% (*n* = 134) of our sample had greater diabetes-related emotional distress. The main demographic and clinical features according to this categorization are shown in Table 1. As can be seen, individuals with higher emotional distress were younger (*d* = 0.49), with shorter illness evolution (*d* = 0.26), reported more hepatic diseases as comorbidities and had a confirmed diagnosis of major depression.

### 3.2. Health-Related Quality of Life

**Figure 1:**
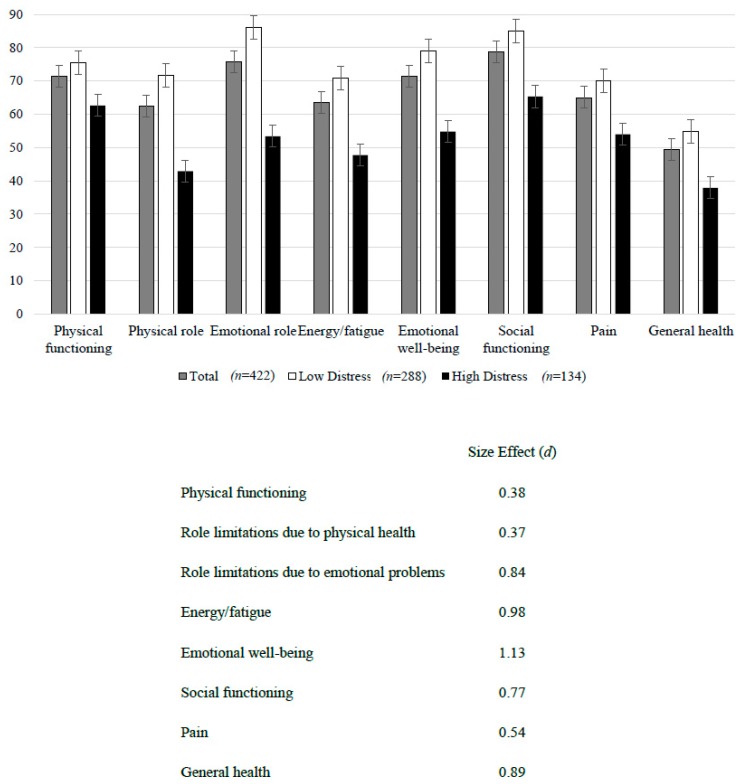
Health-related quality of life dimensions according to emotional distress status.

The eight dimensions of health-related quality of life assessed with the SF-36 showed that DM2 patients had moderate difficulties in social functioning, role limitations due to emotional problems, physical functioning, and emotional well-being (scores over 70), with more difficulties in the dimension of pain, energy/fatigue and role limitations due to physical health (scores between 61–66), as well as important difficulties in general health (score < 50). Patients with high emotional distress reported more difficulties than those with low emotional distress (*p* < 0.001 in all dimensions), all with a moderate to high effect size, with the exception of physical functioning with a low effect size (see Figure 1).

## 4. Discussion

Many studies have evaluated the association between type 2 diabetes mellitus, depression and quality of life, but few have focused on studying how living with emotional distress can influence the quality of life in Mexican individuals with type 2 diabetes mellitus [6,21]. Therefore, the aim of the present study was to compare demographic features, diabetes clinical characteristics and quality of life between patients with high and low diabetes-related emotional distress.

We observed that the presence of depression and hepatic injury were the main factors associated with high diabetes-related emotional-distress. Our results are consistent with previous reports that show that individuals with type 2 diabetes mellitus develop major depression and complications such as hepatic diseases [21,22,23]. According to Sánchez Cruz et al. [21] stress is responsible for uncontrolled glycemia in individuals with DM2, poor adherence to treatment and major depression [21]. These data are consistent with our results, as the majority of our participants with high levels of distress also had higher levels of depression. Also, the literature has described that individuals with diabetes and depression are more likely to use health care services, use multiple medications and exhibit a higher number of comorbid medical conditions. In consequence, they live with high diabetes related emotional-distress as was observed in the present study [24]. The differences observed in individuals with high and low diabetes-related emotional-distress are relevant as they seemed to be non-related to demographic features or any other variables (such as body mass index), features that have been previously described as related to emotional distress [25]. Early detection and intervention of individuals with diabetes mellitus are a priority for preventing diabetes-related emotional-distress, medical comorbidities and psychiatric disorders such as depression.

High distress due to living with diabetes plays an important role in individuals with diabetes mellitus [6]. For instance, we observed low quality of life in all subscales in patients with high distress; additionally, the quality of life in this group was significantly lower than in low diabetes-related emotional distress individuals. The lowest scores were observed in the areas of physical role and general health. This could be partially due to the age of the participants, for the mean was above 50 years old and fewer years of illness, while older patients had had a prolonged illness evolution. Therefore, younger patients may exhibit more comorbidities than older patients [24]. It is also possible that these younger patients were reluctant or unable to follow the treatment, for medical comorbidities make it difficult to follow a restrict diet or exercise regularly, which could be the case in our participants, as a high proportion showed poor glycemic control [26]. Then, it could be hypothesized that poor glycemic control generates emotional stress in younger patients. Furthermore, we observed in our participants that emotional stress was indirectly linked to quality of life; considering that high distress itself could cause patients with diabetes to perceive this disease negatively, emotional distress will have an even greater impact on the control and treatment of diabetes. This cycle is detrimental to their health, as clinical improvement becomes a growing challenge causing greater emotional stress in patients with diabetes. Therefore, we could assume that older patients with a prolonged illness evolution are more likely to follow the appropriate treatment and care due to their knowledge and familiarity with the disease.

There are some explanations of the biological mechanism of emotional distress and type 2 diabetes mellitus. First, it is well known that chronic psychological stress is associated with depression and also with type 2 diabetes mellitus [27]. A well-supported theory of type 2 diabetes mellitus involves increased levels of adrenocorticotropic hormone (ACTH) promoting the activity of the hypothalamic-pituitary-adrenal (HPA) axis, the axis itself modifies glucose levels by feeding back the vicious circle [28]. Furthermore, HPA is a key mediator of the stress response regulating the secretion of glucocorticoids by the adrenal gland, which participates in several endocrine and neuropsychiatric diseases [29]. Therefore, brain insulin signaling dysfunction could impair the HPA axis normal response to stress, possibly facilitating the development of depression for example [30]. Furthermore, blood glucose is known to affect mood; inversely, depression has been suggested to be a possible cause of inadequate metabolic control in patients with type 2 diabetes mellitus [26].

A second possible explanation is that inflammation biomarkers are elevated by chronic inflammation such as TNF alpha and interleukins. Chronic inflammation may play an integral role in the relationship between diabetes-stress and depression, given the role it has in chronic illnesses. For example, TNFα stimulates blood-brain barrier (BBB) disruption in patients with type 2 diabetes mellitus, which could ultimately lead to loss of the BBB transport regulation of other inflammatory signals, and exacerbate allostatic load to the HPA axis, which could also lead to its dysregulation [31,32]. Another point is that TNFα promotes insulin resistance through the phosphorylation of insulin receptor substrate 1 via the activation of cellular stress-responses kinases [33,34]. Moreover, TNFα also activates the nuclear factor κB, which is a transcriptional factor involved in neuronal survival and transcription rate of other cytokines, that will cause insulin signaling impairment [35]. We could assume that a pathway involving TNFα and insulin resistance could be a mechanistic link between T2D and depression, for example. These biological mechanisms, as we have mentioned, involve complications, from dysregulations at glucose level, to hepatic, renal, neuropathic and psychiatric complications such as depression and anxiety [36,37].

Some limitations of this study should be considered. First, there is an overrepresentation of women in our sample and our results should not be generalized to both genders as we did not consider the particular environmental factors that could be influenced by biological sex. Second, we did not address the influence of genetic stratification, even though the state of Tabasco is a highly homogenous state of Mexico in which the majority population is native to the place. Additionally, we did not address the possible participation of education level and/or socioeconomic status in the triad quality of life-emotional distress-type 2 diabetes mellitus.

## 5. Conclusions

Despite the limitations, our results evidence the importance and impact of emotional distress during the course of diabetes mellitus and its impact on the quality of life of these patients. Future studies should have a longitudinal evaluation of this phenomenon including patients with shorter illness evolution to set the basis for the implementation of specific interventions targeted to reduce distress in this population. Individuals with diabetes should receive multidisciplinary treatment, aiming to improve treatment adherence, physical and mental health, giving patients the opportunity to achieve life goals with a better quality of life. Health professionals have a commitment to patients and should play an active role in their assessment and treatment. A multidisciplinary team may improve the patient’s quality of life by reducing emotional distress and may even aim to reduce the incidence of this disease in our population.

## Figures and Tables

**Table 1:** Demographic and clinical features according to emotional distress status.

	Total Sample *n* = 422		Low Distress *n* = 288		High Distress *n* = 134		Statistics
	*n*	*%*	*n*	*%*	*n*	*%*	
Gender-Women	294	69.7	195	67.7	99	73.9	Fisher = 0.19
Marital status-Married	258	61.1	184	63.9	74	66.2	Fisher = 0.10
Socioeconomic status							Fisher = 0.20
Medium	187	44.3	134	46.5	53	39.6	
Low	231	54.7	151	52.4	80	59.7	
Non-remunerated activity	280	66.4	184	63.9	96	71.6	Fisher = 0.12
BMI—Overweight/obesity	333	78.9	235	81.6	98	73.1	Fisher = 0.06
HbA1C ≥ 6.5	340	80.6	227	78.8	113	84.3	Fisher = 0.18
Medical comorbidity							
-Hepatic	54	12.8	28	9.7	26	19.4	Fisher = 0.008
-Renal	103	24.4	65	22.6	38	28.4	Fisher = 0.22
-Neuropathy	191	45.3	125	43.4	66	49.3	Fisher = 0.29
-Cardiovascular	184	43.6	124	43.1	60	44.8	Fisher = 0.74
Current treatment							
-Insulin	296	70.1	194	67.4	102	76.1	Fisher = 0.07
-Hypoglycemic agent	387	91.7	269	93.4	118	88.1	Fisher = 0.08
-Antihypertensive agent	203	48.1	142	49.3	61	45.5	Fisher = 0.53
Substance use							
-Alcohol—Yes	67	15.9	43	14.9	24	17.9	Fisher = 0.43
-Marihuana—Yes	2	0.5	1	0.3	1	0.7	Fisher = 0.53
-Nicotine—Yes	23	5.5	17	5.9	6	4.5	Fisher = 0.65
Psychiatric comorbidity							
-Major depression—Yes	46	10.9	25	8.7	21	15.7	Fisher = 0.04
-Manic episodes—Yes	1	0.2	1	0.3	-		Fisher = 1.00
-Alcohol use disorder—Yes	2	0.5	1	0.3	1	0.7	Fisher = 0.53
-Psychosis—Yes	1	0.2	1	0.3	-		Fisher = 1.00
	Mean	S.D.	Mean	S.D.	Mean	S.D.	
Current age	54.8	12.8	56.8	12.1	50.6	13.1	t =1.2, *p* = 0.23
Years of education	6.9	4.4	7.1	4.5	6.6	4.4	t = 0.2, *p* = 0.83
Illness evolution (years)	15.1	8.7	15.8	8.8	13.6	8.1	t = 0.2, *p* = 0.82

## References

[1] Cho N., Shaw J., Karuranga S., Huang Y., da Rocha Fernandes J., Ohlrogge A., Malanda B. (2018). IDF Diabetes Atlas: Global estimates of diabetes prevalence for 2017 and projections for 2045. Diabetes Res. Clin. Prac..

[2] Federation I.D. (2015). IDF Diabetes Atlas.

[3] (2017). Global, regional, and national incidence, prevalence, and years lived with disability for 328 diseases and injuries for 195 countries, 1990–2016: A systematic analysis for the Global Burden of Disease Study 2016. Lancet.

[4] DeFronzo R.A., Ferrannini E., Alberti K.G.M.M., Zimmet P., Alberti G. (2015). International Textbook of Diabetes Mellitus, 2 Volume Set.

[5] Molina Iriarte A., Acevedo Giles O., Yáñez Sandoval M.E., Dávila Mendoza R., González Pedraza Avilés A. (2013). Comparación de las prevalencias de duelo, depresión y calidad de vida asociados con la enfermedad entre pacientes con diabetes mellitus tipo 2 descontrolados y controlados. Rev. Espec. Médico-Quirúrgicas.

[6] Juárez-Rojop I.E., Fortuny-Falconi C.M., González-Castro T.B., Tovilla-Zárate C.A., Villar-Soto M., Sanchez E.R., Hernández-Díaz Y., López-Narvaez M.L., Ble-Castillo J.L., Pérez-Hernández N. (2018). Association between reduced quality of life and depression in patients with type 2 diabetes mellitus: A cohort study in a Mexican population. Neuropsychiatr. Dis. Treat..

[7] Ogurtsova K., da Rocha Fernandes J., Huang Y., Linnenkamp U., Guariguata L., Cho N., Cavan D., Shaw J., Makaroff L. (2017). IDF Diabetes Atlas: Global estimates for the prevalence of diabetes for 2015 and 2040. Diabetes Res. Clin. Prac..

[8] Rivera-Ledesma A., Lena M.M.-L., Sandoval-Ávila R. (2012). Desajuste psicológico, calidad de vida y afrontamiento en pacientes diabéticos con insuficiencia renal crónica en diálisis peritoneal. Salud Ment..

[9] Granados E.E., Escalante E. (2010). Estilos de personalidad y adherencia al tratamiento en pacientes con diabetes mellitus. Liberabit.

[10] Lazcano Ortiz M., Salazar González B.C. (2007). Estrés percibido y adaptación en pacientes con diabetes mellitus tipo 2. Aquichan.

[11] Ortiz M., Ortiz E., Gatica A., Gómez D. (2011). Factores psicosociales asociados a la adherencia al tratamiento de la diabetes mellitus tipo 2. Ter. Psicol..

[12] Schmidt C.B., van Loon B.J.P., Vergouwen A.C.M., Snoek F.J., Honig A. (2018). Systematic review and meta-analysis of psychological interventions in people with diabetes and elevated diabetes-distress. Diabet. Med..

[13] Babenko A.Y., Mosikian A.A., Lebedev D.L., Khrabrova E.A., Shlyakhto E.V. (2019). Mental state, psychoemotional status, quality of life and treatment compliance in patients with Type 2 diabetes mellitus. J. Comp. Eff. Res..

[14] Siqueira P., Dos Santos M., Zanetti M., Ferronato A. (2007). Dificultades de los pacientes diabéticos para el control de la enfermedad: Sentimientos y comportamientos. Rev. Latino-Am. Enferm..

[15] Iriarte A.M., Acevedo Giles O., Sandoval M.E.Y., Dávila Mendoza R., González Pedraza Avilés A. (2013). Comparison of prevalence of mourning, depression and quality of life related to disease between patients with uncontrolled and controlled diabetes mellitus type 2. Rev. Espec. Médico-Quirúrgicas.

[16] Organization W.H. (2011). Use of Glycated Haemoglobin (HbA1c) in Diagnosis of Diabetes Mellitus: Abbreviated Report of a WHO Consultation.

[17] Durán-Arenas L., Gallegos-Carrillo K., Salinas-Escudero G., Martínez-Salgado H. (2004). Towards a Mexican normative standard for measurement of the short format 36 health-related quality of life instrument. Salud Publica Mex..

[18] Zúniga M.A., Carrillo-Jiménez G.T., Fos P.J., Gandek B., Medina-Moreno M.R. (1999). Evaluación del estado de salud con la Encuesta SF-36: Resultados preliminares en México. Salud Pública Méx..

[19] McGuire B., Morrison T., Hermanns N., Skovlund S., Eldrup E., Gagliardino J., Kokoszka A., Matthews D., Pibernik-Okanović M., Rodríguez-Saldaña J. (2010). Short-form measures of diabetes-related emotional distress: The Problem Areas in Diabetes Scale (PAID)-5 and PAID-1. Diabetologia.

[20] Cohen J. (2013). Statistical Power Analysis for the Behavioral Sciences.

[21] Sánchez-Cruz J.F., Hipólito-Lóenzo A., Mugártegui-Sánchez S.G., Yáñez-González R.M. (2016). Estrés y Depresión asociados a la no adherencia al tratamiento en pacientes con Diabetes Mellitus tipo 2. Atención Fam..

[22] Viveros G.R.O., Herrera É.O. (2011). Capacidad predictiva de la adherencia al tratamiento en los modelos socio-cognitivos de creencias en salud. Psicol. Y Salud.

[23] Pedraza Banderas G.L., Vega Valero C.Z. (2019). Caracterización psicosocial de pacientes diabéticos mexicanos. Rev. Electrón. Psicol. Iztacala.

[24] Le T.K., Curtis B., Kahle-Wrobleski K., Johnston J., Haldane D., Melfi C. (2011). Treatment patterns and resource use among patients with comorbid diabetes mellitus and major depressive disorder. J. Med. Econ..

[25] Bruno B.A., Choi D., Thorpe K.E., Yu C.H. (2019). Relationship Among Diabetes Distress, Decisional Conflict, Quality of Life, and Patient Perception of Chronic Illness Care in a Cohort of Patients with Type 2 Diabetes and Other Comorbidities. Diabetes Care.

[26] Anderson B.J., McKay S.V. (2011). Barriers to glycemic control in youth with type 1 diabetes and type 2 diabetes. Pediatr. Diabetes.

[27] Chan O., Inouye K., Akirav E., Park E., Riddell M.C., Vranic M., Matthews S.G. (2005). Insulin alone increases hypothalamo-pituitary-adrenal activity, and diabetes lowers peak stress responses. Endocrinology.

[28] Chong A.C., Vogt M.C., Hill A.S., Bruning J.C., Zeltser L.M. (2015). Central insulin signaling modulates hypothalamus-pituitary-adrenal axis responsiveness. Mol. Metab..

[29] Lopez J.F., Chalmers D.T., Little K.Y., Watson S.J.A.E. (1998). Bennett Research Award. Regulation of serotonin1A, glucocorticoid, and mineralocorticoid receptor in rat and human hippocampus: Implications for the neurobiology of depression. Biol. Psychiatry.

[30] Lyra E.S.N.M., Lam M.P., Soares C.N., Munoz D.P., Milev R., De Felice F.G. (2019). Insulin Resistance as a Shared Pathogenic Mechanism Between Depression and Type 2 Diabetes. Front. Psychiatry.

[31] Starr J.M., Wardlaw J., Ferguson K., MacLullich A., Deary I.J., Marshall I. (2003). Increased blood-brain barrier permeability in type II diabetes demonstrated by gadolinium magnetic resonance imaging. J. Neurol. Neurosurg. Psychiatry.

[32] Felger J.C., Haroon E., Woolwine B.J., Raison C.L., Miller A.H. (2016). Interferon-alpha-induced inflammation is associated with reduced glucocorticoid negative feedback sensitivity and depression in patients with hepatitis C virus. Physiol. Behav..

[33] Clarke J.R., Lyra E.S.N.M., Figueiredo C.P., Frozza R.L., Ledo J.H., Beckman D., Katashima C.K., Razolli D., Carvalho B.M., Frazao R. (2015). Alzheimer-associated Abeta oligomers impact the central nervous system to induce peripheral metabolic deregulation. EMBO Mol. Med..

[34] Lourenco M.V., Clarke J.R., Frozza R.L., Bomfim T.R., Forny-Germano L., Batista A.F., Sathler L.B., Brito-Moreira J., Amaral O.B., Silva C.A. (2013). TNF-alpha mediates PKR-dependent memory impairment and brain IRS-1 inhibition induced by Alzheimer’s beta-amyloid oligomers in mice and monkeys. Cell Metab..

[35] Gupta S., Bi R., Kim C., Chiplunkar S., Yel L., Gollapudi S. (2005). Role of NF-kappaB signaling pathway in increased tumor necrosis factor-alpha-induced apoptosis of lymphocytes in aged humans. Cell Death Differ..

[36] Dantzer R., O’Connor J.C., Freund G.G., Johnson R.W., Kelley K.W. (2008). From inflammation to sickness and depression: When the immune system subjugates the brain. Nat. Rev. Neurosci..

[37] Li J., Sun X., Yu Y. (2013). The prevalence of impaired glucose regulation in psychiatric patients with sleep disorders and its relationship with altered hypothalamopituitary–adrenal and hypothalamopituitary–thyroid axis activity. Sleep Med..

